# An inconclusive study comparing the effect of concrete and abstract descriptions of belief-inconsistent information

**DOI:** 10.1371/journal.pone.0189570

**Published:** 2018-02-15

**Authors:** Katherine A. Collins, Richard Clément

**Affiliations:** School of Psychology, University of Ottawa, Ottawa, Ontario, Canada; Waseda University, JAPAN

## Abstract

Linguistic bias is the differential use of linguistic abstraction (as defined by the Linguistic Category Model) to describe the same behaviour for members of different groups. Essentially, it is the tendency to use concrete language for belief-inconsistent behaviours and abstract language for belief-consistent behaviours. Having found that linguistic bias is produced without intention or awareness in many contexts, researchers argue that linguistic bias reflects, reinforces, and transmits pre-existing beliefs, thus playing a role in belief maintenance. Based on the Linguistic Category Model, this assumes that concrete descriptions reduce the impact of belief-inconsistent behaviours while abstract descriptions maximize the impact of belief-consistent behaviours. However, a key study by Geschke, Sassenberg, Ruhrmann, and Sommer [2007] found that concrete descriptions of belief-inconsistent behaviours actually had a greater impact than abstract descriptions, a finding that does not fit easily within the linguistic bias paradigm. Abstract descriptions (e.g. *the elderly woman is athletic*) are, by definition, more open to interpretation than concrete descriptions (e.g. *the elderly woman works out regularly*). It is thus possible that abstract descriptions are (1) perceived as having less evidentiary strength than concrete descriptions, and (2) understood in context (i.e. athletic *for an elderly woman*). In this study, the design of Geschke et al. [2007] was modified to address this possibility. We expected that the differences in the impact of concrete and abstract descriptions would be reduced or reversed, but instead we found that differences were largely absent. This study did not support the findings of Geschke et al. [2007] or the linguistic bias paradigm. We encourage further attempts to understand the strong effect of concrete descriptions for belief-inconsistent behaviour.

## Introduction

It has long been accepted that beliefs shape language use both intentionally and unintentionally, resulting in biased language [1, 2]. The unintentional expression of biased beliefs has been found within many different contexts [3, 4] and previous research has demonstrated that it occurs spontaneously (e.g. [5, 6]) and without awareness [6, 7]. It can take different forms, such as the negation bias [8] and the irony bias [9], but most of the research is on the linguistic intergroup bias (lib; [10]) and the linguistic expectancy bias (leb; [11]), both of which describe the differential use of linguistic abstraction for members of different groups. According to this paradigm, not only do speakers reveal their true beliefs by expressing linguistic bias but these beliefs are also transferred to recipients. Previous research has, however, been inconclusive on whether abstract or concrete words have a stronger impact on recipients for belief-inconsistent information. Thus, the focus of the current study was to investigate the effect of linguistic abstraction when communicating belief-inconsistent information.

Linguistic abstraction is usually operationalized through the Linguistic Category Model (lcm; [12]), which was built out of attribution research on implicit verb causality (e.g. [13, 14]). The central premise behind the lcm is that different word categories (verbs, adjectives, etc.) have different cognitive implications. The model thus distinguishes between four word categories that vary in the amount of abstraction required to describe behaviour in such terms. The four categories, from concrete to abstract, are descriptive action verbs (davs), interpretive action verbs (iavs), state verbs (svs), and adjectives (adjs). As an example, imagine that you have witnessed the following behaviour: *John hugs Dawn*. This could be described as it is now with a dav (*hugs*), or with an iav (*John comforts Dawn*), sv (*John loves Dawn*), or adj (*John is affectionate*). Any of these descriptions are correct, but as linguistic abstraction increases there is a greater degree of interpretation and less specific information about the physical aspects of the behaviour.

descriptive action verbs typically refer to one physically invariant aspect of the behaviour (hugging always involves wrapping arms around somebody) and are basically a description of the behaviour itself. iavs describe a class of behaviours: *comforts*, for example, can refer to several davs since there are many ways to offer comfort. svs pertain to the mental state of the person performing the behaviour, an aspect that is not directly observable or verifiable. And adjs, the most abstract word category, correspond to a trait of the person performing the behaviour. By referring to a trait, adjs go beyond the specific details of the behaviour and generalize across time and situations: Someone who is affectionate will likely hug other people in the future and will also likely engage in other affectionate behaviours even outside the context of offering comfort. Thus, the different levels of linguistic abstraction differ in the amount of interpretation and generalizing that occurs from the behaviour.

The lib is the tendency to use concrete words to describe negative ingroup and positive outgroup behaviours, while using abstract words to describe positive ingroup and negative outgroup behaviours. Concrete words will limit the impact of the behaviour to the specific circumstances in which it took place, while abstract words generalize the impact of the behaviour across time and situations. Use of this bias thus implies that the speaker expects socially undesirable (negative), but not socially desirable (positive), behaviour from outgroups and socially desirable, but not socially undesirable, behaviour from the ingroup. It is for this reason that researchers who employ this paradigm have argued that linguistic bias is a mechanism for transmitting biased beliefs to, and maintaining biased beliefs of, recipients [15, 16]. This argument remains only an assumption, however, until research on the consequences of linguistic bias can demonstrate that exposure has the expected effects.

Though many authors have noted the dearth of research on the consequences of biased language exposure [17, 2, 18], only recently have they started to address it. These studies have shown that exposure to biased language affects recipients’ impression of the described individual [19–21], attitude towards the social group to which the described individual belongs [22, 23], perception of the speaker [24–27], and perception of the interpersonal distance between the recipient and speaker [28]. The effect of linguistic bias is fundamentally due to the impact of low and high levels of linguistic abstraction.

In a previous study [19], we systematically manipulated linguistic abstraction to demonstrate a causal effect on person impression formation. Higher levels of linguistic abstraction increased the stereotypicality of the impression and the strength of dispositional attributions. Notably, the nature of the communicative context influenced the reception of linguistic abstraction in that socially desirable and undesirable behaviours resulted in more extreme evaluations in intergroup situations than in intragroup situations. This study provided support for the theoretical argument that linguistic bias plays a role in belief transmission, with the caveat that reception is also a function of the communicative context. This previous study did not, however, consider typicality—that some traits or behaviours might be considered more typical of some groups versus others. Behaviours were either socially desirable or undesirable and not more typical of either the ingroup or outgroup members that were described. Yet, if linguistic bias plays a role in belief maintenance, then the typicality of behaviours and its congruency with pre-existing beliefs is important.

Belief-inconsistent behaviours, in particular, play an essential role since they must be discounted or explained in order for beliefs to be maintained. Within the linguistic bias paradigm, concrete descriptions are generally used to communicate belief-inconsistent information and it is assumed, according to the lcm, that abstract information has a greater impact on recipients than does concrete information. By extension, even repeated exposure to information described at concrete levels of linguistic abstraction should not have an impact on the perception or beliefs of recipients. There is some research to support this idea. Research on person perception, for example, has found that more abstract descriptions do have a stronger impact on recipients (e.g. [21]). Geschke et al. [23] also found that abstractly worded news articles led to a larger estimate of criminal behaviour and stronger subtle (but not blatant) prejudice than concretely worded news articles. Further, Gorham [29] found, as expected, that people who are heavy consumers of media use more abstract language for a Black target in a crime story whereas light consumers do not, suggesting that recipients exposed to linguistic bias are more likely to use linguistic bias. This is, however, generally within the context of belief-consistent information.

In the context of belief-inconsistent information, and in contrast with the linguistic bias framework, there is compelling evidence that concrete messages actually have a greater impact on recipients than abstract messages. This makes intuitive sense because concrete descriptions give more details about the behavioural event, are more easily verified, and less open to interpretation. Thus concrete descriptions could have more evidentiary value than abstract descriptions, which involve fewer specific details about the behaviour and more interpretation. In support of this, researchers in cognitive processing have found evidence for what is termed a concreteness effect. Ter Doest, Semin, and Sherman [30], for example, found that concrete messages cue deeper processing which leads to more attention to, and recall of, concrete information as compared to abstract information. This study suggests that concretely described information has a greater impact than abstractly described information. Further, Ewell [31], in contrast to Gorham [29], found that news stories did not change implicit attitudes and that the race of the subject of the article did not impact the recipient.

Thus, the currently available evidence is inconclusive on whether it is concretely or abstractly described belief-inconsistent information that has a greater impact on recipients. The linguistic bias paradigm argues that abstract descriptions have a greater impact while some studies show that concrete descriptions have a greater impact. Recall that linguistic bias is, essentially, the description of belief-inconsistent information in concrete terms. The relative impact of concrete and abstract descriptions of belief-inconsistent information is, therefore, important to determining the effects of the prevalent use of linguistic bias. It has the potential for one of two roles in the communication of belief-inconsistent information. First, it can maintain beliefs by limiting the impact of such behaviours, as expected by the linguistic bias paradigm. Or, second, it can change beliefs by increasing the impact of such behaviours, as suggested by ter Doest et al. [30]. The goal of the current study is to clarify the relative impact of abstract and concrete described belief-inconsistent information.

Closest to this goal is a key study by Geschke, Sassenberg, Ruhrmann, and Sommer [32] that was conducted with the specific objective of clarifying what impact concrete information has on recipients. In this study, participants read about a belief-inconsistent exemplar and then answered questions about their impressions. The authors were also interested in varying communication sources but only the relevant portion of the study will be outlined here. The text described the athleticism of a 74-year-old female, named Bruni, who participated in an amateur triathlon. There were two versions of the text, in which the behaviours were either described at a concrete (*she works out regularly*) or abstract (*she is athletic*) level. After a distractor task to avoid word for word recall, participants were asked to estimate the likelihood of a variety of future athletic behaviours (e.g. participating in a bicycle race), rate the athleticism of the exemplar (e.g., *in my opinion*, *Bruni is fit*), and indicate their endorsement of the belief that the elderly are unathletic. It was found that when the target was described using concrete levels of linguistic abstraction, the belief-inconsistent exemplar was perceived as more athletic and more likely to engage in future athletic behaviours. Thus, this study suggests that concrete messages have more impact on recipients than do abstract messages for the communication of belief-inconsistent information.

The findings reported by Geshke et al. [32] do not fit with the data and theory of the linguistic bias paradigm, which argues that the prevalent use of linguistic bias leads to belief maintenance. According to the lcm, prevalent use would lead to belief maintenance since lower levels of linguistic abstraction limit the impact of a behaviour to the specific circumstances in which it took place. Concretely describing belief-inconsistent information, then, allows pre-existing beliefs to remain intact by signalling to recipients that the information can be discounted or ignored. The study by Geschke et al. [32], in contrast, suggests that concrete descriptions of belief-inconsistent information actually have a greater effect than abstract descriptions. This would suggest a corresponding increase in the impact of belief-inconsistent information, potentially leading to a change in pre-existing beliefs. If so, then linguistic bias, and specifically the tendency to concretely describe belief-inconsistent information, would not play a role in belief maintenance but in belief transformation.

How can the results of Geschke et al. [32] be reconciled with the linguistic bias paradigm. From a communication perspective, information that is shared does not need to be communicated because it is already understood to be shared [33, 34]. In the abstract text of this experiment, when the belief-inconsistent exemplar was described as “athletic”, what participants might have understood is that the exemplar was “athletic *for an elderly person*”. The exemplar had already been established as being elderly and, given the lack of detail on what constituted athleticism, the participant may have understood the term “athletic” within this context. That is, there may have been an implicit understanding that the term “athletic” was in comparison to other elderly people. The concrete descriptions in the concrete text, in contrast, allowed the participants to compare the exemplar’s behaviour to all of their previous experience with athletic people and athletic behaviours, and provided evidence that the exemplar was athletic in comparison to the general population—not just elderly people. That is, the abstract descriptions used in the experiment may be perceived as having less evidentiary value for athleticism (for the population in general) than the concrete descriptions. Thus, it cannot be concluded that concrete language and not evidentiary value was responsible for the results. Further, the researchers focused on the interpersonal level of analysis whereas the group level of analysis may be more relevant. It could be that concrete language has more of an effect at the interpersonal level (belief-inconsistent exemplar) but that abstract language has more of an effect at the group level (beliefs about the elderly as unathletic).

The present study used a modified design of Geschke et al. [32] to address these issues. Similar to Geschke at al. [32], participants read about a belief-inconsistent exemplar that was described using at either a low (concrete) or high (abstract) level of abstraction then reported their perception of the exemplar and the behaviour itself. A control condition was also included which presented participants with information concerning the belief-inconsistent exemplar, withholding specific abstract and concrete details. This was done to establish a baseline reaction to the exemplar against which the reactions to the abstractness manipulation could be compared. After reading the text, participants then completed a modified version of the dependent measures used by Geschke et al. [32] in which they were asked to rate the athleticism of the exemplar compared to the general population and to other elderly people. This measure made explicit any information that might have been implicitly understood by participants in the study by Geschke et al. [32]. Given that concrete descriptions have strong evidentiary value and given the results of Geschke at al. [32], we expected the impact of concrete descriptions on recipients’ impression of the exemplar to be high regardless of how the comparison was drawn. On the other hand, from a communication perspective, we expected that abstract descriptions would have a greater impact when the exemplar was compared to other older people, since that is what might be implicitly understood when no further details are given.

Participants were also asked to indicate their endorsement of the belief that the elderly are not athletic. Geschke at al. [32] included this measure but did not analyze whether abstract or concrete information influenced the strength of endorsement. This measure will be analyzed to see if concrete or abstract language has a stronger effect on the perception of the group as a whole, not just at the interpersonal level. We expected that the abstract text will have a greater impact than the concrete text on this measure of group perception.

## Method

This study, including the consent procedure was approved by the Research Ethics Board of the University of Ottawa. The participants signified consent by clicking the appropriate icon at the bottom of the Informed Consent statement. The decision to consent was recorded in the database. Participants were not permitted to continue to the study and give data if they did not consent.

### Participants

There were 133 participants, 98 females and 35 males, all University of Ottawa students who completed the experiment in return for course credit. There were no inclusion or exclusion criteria. The mean age of participants was 19.45 (*SD* = 2.76). The sample was, therefore, similar to Geschke et al’s [32] undergraduate students, mean-aged 22 years with a range of 19 to 28 years.

### Materials

The two original German texts, abstract and concrete, from Geschke et al. [32] were obtained [Geschke D, personal communication, August 2, 2013]. The texts were translated into English then back translated into German by two independent English-German bilingual speakers. The original and back-translated texts were then compared by a third English-German bilingual speaker, who made adjustments so that the meaning of the English translations was comparable to that of the German originals. In the control version of the text, which was not included in Geschke et al.’s [32] design, no additional details about the counter-stereotypical exemplar were given. The three texts are reproduced in the appendix with the sentences distinguishing the concrete and abstract versions bolded.

### Measures

#### Manipulation check

Geschke et al. [32] conducted a pretest to ensure that the exemplar was perceived as unexpected. Instead of a single item, we used three items (*α* = .781) measured on a 7-point Likert scale anchored at 1 (*Strongly Disagree*) and 7 (*Strongly Agree*). Participants had to indicate the extent to which they agreed that the behaviour in the text was unexpected. Items were: (1) *[the exemplar]’s athleticism is surprising* (2) *I find it extraordinary that someone [the exemplar]’s age is so athletic*, and (3) *I would not expect someone like [the exemplar] to engage in sports regularly*. A higher number indicated that the behaviour was perceived as more unexpected.

#### Evidentiary strength of the behaviour

Given our expectation that the abstract and concrete texts might have different evidentiary strength, we measured perception of the behaviour as athletic with one item: *I consider the activities described in the text to be athletic*. This was not measured in Geschke et al. [32]. Participants indicated their agreement on a 7-point Likert scale anchored at 1 (*Strongly Disagree*) and 7 (*Strongly Agree*). A higher number indicated that the behaviour was perceived as more athletic.

#### Perceived athleticism

As in Geschke et al. [32], likelihood of future athletic behaviours had four items (*α* = .839). In contrast to Geschke et al. [32], who used a 0 to 100 scale, these items were measured on a 7-point Likert scale anchored at 1 (*Not at all likely*) and 7 (*Extremely likely*). A higher number indicated a higher likelihood of participating in future athletic behaviours.

Similar to Geschke et al. [32], we also measured perceived athleticism of the exemplar with four items but we modified the questions so that the comparison group was made explicit. Thus, perceived athleticism of the exemplar had eight items, measured on a 7-point Likert scale anchored at 1 (*Totally Disagree*) and 7 (*Totally Agree*). Each item followed the form of “*In my opinion*, *[the exemplar] is [adjective] in comparison to [group]*”. There were four adjectives (*athletic*, *fit*, *weak*, and *unathletic*) and two comparison groups (*the general population*, *other older people*). Cronbach’s alpha was based on four items each for the older comparison group (*α* = .890) and general population comparison group (*α* = .874). Items were reverse coded as necessary so that a higher number indicated more perceived athleticism.

#### Belief endorsement

We measured endorsement of the belief that the elderly are not athletic. Instead of a single item as in Geschke et al. [32], we used four items (*α* = .636), measured on a 7-point Likert scale anchored at 1 (*Strongly Disagree*) and 7 (*Strongly Agree*). Participants had to indicate the extent to which they agreed with different statements about the elderly: (1) *older people are not often athletic* (2) *older people are often frail* (3) *older people often complete in triathlons*, *and* (4) *older people usually compete in racing events*. Items were reverse coded as necessary such that a higher number indicated more endorsement of the belief that elderly people are not athletic.

### Procedure

The procedure was the same as that used in Geschke et al.’s [32] study except for the following modifications: the pretext used for conducting the study, the inclusion of a control group, and changes to the measures outlined above.

Participants signed up for a study believing that they would be evaluating supplementary material from textbooks, such as newspaper articles and links to online videos. They were given access to a secure website, where they could complete the study online at their own convenience.

Participants read the text about a belief-inconsistent exemplar that was ostensibly a news article included in a psychology textbook. Afterwards, they were asked to evaluate the text and author of the text, including some questions on educational value to make the purpose of the study more plausible. Participants also evaluated a short film (~11 minutes) that was portrayed as online supplementary material from a psychology textbook.

Finally, participants were informed that they would answer questions about either the text or the video to assess their comprehension. They were asked to report what they had learned and not to repeat what the text or video had expressed. These questions assessed the participants’ impression of the person described and of elderly people in general. Participants also answered some demographic questions and were debriefed at the end of the study.

## Results

The alpha level was set at .05 for all omnibus tests and .01 for all post-hoc tests to adjust for multiple comparisons.

### Manipulation check

Because it is possible that exposure to the abstract and concrete texts could influence participants’ perception of the behaviour and belief endorsement, only the data from participants who were exposed to the control text were analyzed. The mean perceived unexpectedness (*M* = 4.77, *SD* = 1,046) was significantly higher than the midpoint of the scale (*t* (37) = 4,550, *p <* 0.001, *d =* .738), indicating that the exemplar was indeed perceived as unexpected as was found in Geschke et al. [32].

### Evidentiary strength of behaviour

The one-way between-subjects analysis of variance of Exposure (Concrete, Abstract, or Control text) on perception of the behaviour as athletic produced a marginally significant effect (*F* (2, 129) = 2,642, *p =* 0.075, *η*_*p*_^*2*^ *=* .039]. Distributions of means and standard deviations [Table 1] suggested that the difference was due to lower athletic ratings from participants in the Control condition. Thus, a contrast was conducted between Control versus the Concrete and Abstract conditions. The contrast was only marginally significant (*F* (1, 129) = 5,130, *p =* 0.025, *η*_*p*_^*2*^ *=* .038) when applying the set cutoff to post-hoc tests.

**Table 1:** Means and standard deviations for perception of the behaviour as athletic.

	Athletic	
Text	Mean	SD
Control	5.24_a_	1,283
Concrete	5.72_b_	1,341
Abstract	5.84_b_	1,098

Subscripts show contrast.

### Perceived athleticism

In contrast to Geschke et al. [32], the one-way between-subjects analysis of variance of Exposure (Concrete, Abstract, or Control text) on the likelihood of future athletic behaviours produced no significant group difference (*F* (2, 129) = 2,016, *p =* 0.137, *η*_*p*_^*2*^ *=* .030).

We further conducted a 3 (Exposure: Concrete, Abstract, or Control) x 2 (Comparison: General Population and Older People) mixed analysis of variance on perception of the exemplar as athletic, with Comparison as the within-subjects factor. There was a significant main effect of Exposure (*F* (2, 129) = 4,426, *p =* 0.014, *η*_*p*_^*2*^ *=* .064), such that the control had lower ratings of athleticism (*M* = 5.18, *SD* = 1,067) than both the concrete (*M* = 5.76, *SD* = 1,064) and abstract (*M* = 5.72, *SD* = 1,067) conditions. There was also a significant main effect of Comparison (*F* (1, 129) = 78,501, *p <*0.001, *η*_*p*_^*2*^ *=* .378), such that the exemplar was perceived as more athletic when compared to other older people (*M* = 5.88, *SD* = 1,114) than when compared to the general population (*M* = 5.23, *SD* = 1,018).

**Fig 1:**
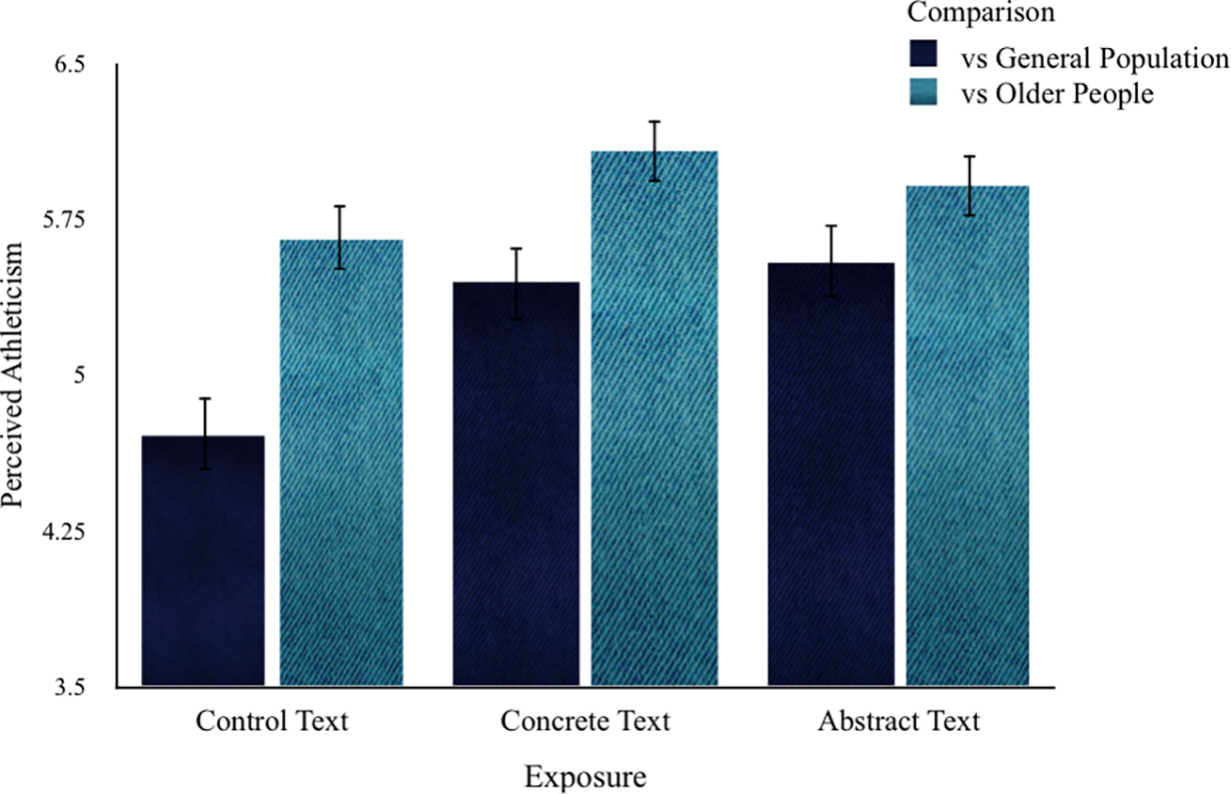
Perceived athleticism as a function of exposure and comparison. Error bars represent standard error.

These effects were, however, subsumed under an interaction between the two factors (*F* (2, 129) = 4,947, *p =* 0.009, *η*_*p*_^*2*^ *=* .071; see Fig 1). Simple main effects were computed in order to delineate its characteristics. Exposure had a significant simple main effect on perception of the exemplar as athletic for the comparison to the general population (*F* (2, 129) = 8,061, *p =* 0.001, *η*_*p*_^*2*^ *=* .111), but not for the comparison to other older people (*F* (2, 129) = 1,549, *p =* 0.216, *η*_*p*_^*2*^ *=* .023). In the case of the comparison to the general population, simple comparisons revealed that the Control condition had lower ratings of athleticism (*M* = 4.71, *SD* = 1,034) than the Concrete (*M* = 5.44, *SD* = 1,005; *p =* 0.001) or Abstract (*M* = 5.55, *SD* = 1,016; *p <*0.001) conditions. The difference between concrete and abstract conditions was not significant (*p =* 0.617). Comparison had a significant simple main effect on perception of the exemplar as athletic for the concrete (*F* (1, 129) = 29,161, *p <*0.001, *η*_*p*_^*2*^ *=* .184), abstract (*F* (1, 129) = 8,284, *p =* 0.005, *η*_*p*_^*2*^ *=* .060), and control (*F* (1, 129) = 48,562, *p <*0,001, *η*_*p*_^*2*^ *=* .273) conditions, resulting in all cases in higher ratings for the comparison to the older people than to the general population.

### Belief endorsement

We analyzed mean endorsement ratings to investigate whether concrete or abstract texts have a different impact on group level beliefs. Geschke et al. [32] did not analyze this measure. We conducted a one-way between-subjects analysis of variance for the effect of Exposure (Concrete, Abstract, or Control text) on endorsement of the belief that older people are not athletic and found no difference across groups (*F* (2, 129) = .263, *p =* 0.769, *η*_*p*_^*2*^ *=* .004). Furthermore, one-sample t-tests comparing the means obtained for the Concrete (M = 4.85, SD = .79), Abstract (M = 4.85, SD = .79) and Control (M = 4.73, SD = .99) conditions to the mid-point of the scale (4) were conducted. Results show that, in each case, participants tended to endorse the belief that older people are not athletic (*t* (49) = 7,606, *p* < .001, *δ* = 1.0759*; t* (43) = 7.196, *p* < .001, *δ* = 1.0759*; t* (37) = 4,553, *p* < .001, *δ* = 0.7374), respectively.

## Discussion

In this study we slightly modified Geschke et al.’s [32] design to ensure that (1) the comparison group was made explicit (2) group level perceptions were analyzed, and (3) a control group was included.

We expected that these modifications would cause the differences in abstract and concrete descriptions to be reversed or reduced. Instead we found that, in most cases, differences were completely eliminated. Being exposed to the belief-inconsistent exemplar, regardless of text, had no impact on endorsement of the belief that the elderly are athletic: Participants exposed to these texts were no more or less likely than the control group to endorse the belief that older people are not athletic. Participants exposed to the concrete and abstract texts also made equal estimates of future athletic behaviour. And, notably, both the concrete and abstract texts were perceived as having equal evidentiary strength, as measured by perception of the behaviour as athletic. In summary, we did not replicate Geschke et al. [32]’s findings.

We did, however, find an expected interaction between Exposure and Comparison, though it was not in the hypothesized direction. Perceived athleticism was high, regardless of text exposure, when the comparison was made to older people but, when the comparison was made to the general population, both abstract and concrete exposures increased perceived athleticism relative to the control condition. This suggests that our belief that the differences reported by Geschke et al. [32] might be due to the comparison group is at least partially substantiated. Given the nature of the control task, the effect can only be attributed, at this point, to more personal details being given about the exemplar in the experimental conditions when the comparison is open to the general population.

The specific comparison to older people may have been subject to a ceiling effect. As shown, participants endorsed the belief that older people are not athletic. The standard or evidence required to be “athletic” in comparison to older people is therefore lower than it is for the comparison to the general population, which could explain why no differences were found between exposures for this condition. Participants presumably had, however, no belief that the general population, which includes many different groups and range of abilities, would be either athletic or not athletic. Thus, there was no ceiling effect for this comparison resulting in higher perceived athleticism for both the abstract and concrete conditions relative to the control condition.

In summary, this study did not support the findings of Geschke et al. [32] or the linguistic bias paradigm. Our design modifications completely and unexpectedly eliminated any differences between the abstract and concrete text. There is no reason to expect that concrete and abstract descriptions would have the same impact. It is possible that the original German text simply did not translate into an appropriate English text. Perhaps the event and details should have been changed to reflect a more Canadian context and the protagonist should have been explicitly portrayed as an ingroup member. It could also be that, combined with the modifications we made to the design, the measures were not sensitive enough to capture the impact of the texts. Our more substantive evidence, limited as it is, calls for more attention being given to reference groups and norms to better understand the attitudinal impact of descriptions. Finally, while acknowledging that this is but one study, we feel that it buttresses the argument in favour of a renewed examination of the processes pertaining to speech abstraction.

## Appendix

### Concrete text

Brunhilde (Bruni) Schukowski from Eberswalde is 74 years old and already a granny three times over. **Neighbors and friends know that, despite her age, she still often engages in sports**. Last weekend she participated in the Brandenburg People’s Triathlon. She was the oldest of the 145 amateur athletes. The event slogan this year was “Young and Old: Active together” and it was organized by city representatives as well as the Charitas. The competition was also a fundraiser for the newly opened Multigenerational Housing Project “We live together” where Bruni resides.

This example shows that older people can also be active. This year, says Bruni, several women and men over 50 participated. In this triathlon “only” the People’s Distance was covered. This is a much shorter distance than that of the professional triathlons. So Bruni had to swim 250 meters, bike 10 kilometers and run 3 kilometers. The first competitors to cross the finish line took about one hour. **Bruni came in 85th with a time of under 2 hours,** **finishing well** **before the last ones.** She was enthusiastically welcomed by her children, grandchildren and neighbors.

A few years ago, while she ran her first triathlon, Bruni wasn’t sure if she would make it, given her age. **While she used to do sports regularly**, it had become increasingly difficult in recent years. Former colleagues, who were noticeably younger, often asked her whether she would participate in the triathlon. And because her participation would be good advertisement for the charitable “Living Together Project”, she finally decided to do it. **Bruni definitely wants to continue with this sport as long as she feels physically well**, she has fun with it and doesn’t find it at all extreme. Well, old people just aren’t what they used to be…“

### Abstract text

Brunhilde (Bruni) Schukowski from Eberswalde is 74 years old and already a granny three times over. **Neighbors and friends know that despite her age, she is still very athletic**. Last weekend she participated in the Brandenburg People’s Triathlon. **For some time now, triathlons have been her favourite sport.** She was the oldest of the 145 amateur athletes. The event slogan this year was ‘Young and Old: Active together’ and it was organized by city representatives as well as the Charitas. The competition was also a fundraiser for the newly opened Multigenerational Housing Project ‘We live together’ where Bruni resides.

This example shows that older people are becoming increasingly active. This year, says Bruni, several women and men over 50 were at the start line. **More and more older people are participating in this sport.** In this triathlon ‘only “the People’s Distance was covered. This is a much shorter distance than that of the professional triathlons. So, Bruni had to swim 250 meters, bike 10 kilometers and run 3 kilometers. The first competitors to cross the finish line took about one hour. **As always**, **Bruni** **finished way** **before the last ones.** She came in 85th, with a time of just under two hours. She was enthusiastically welcomed by her children, grandchildren and neighbors.

A few years ago, while she ran her first triathlon, Bruni wasn’t sure if she would make it, given her age. **While she used to be an active athlete**, it had become increasingly difficult in recent years. Former colleagues, who were noticeably younger, often asked her whether she would participate in the triathlon. And because her participation would be good advertisement for the charitable “Living Together Project”, she finally decided to do it. As long as she continues to feel good physically, **Bruni definitely wants to continue being an active triathlete**, she has fun with it and doesn’t find it at all extreme. Well, old people, just aren’t what they used to be…“

### Control text

Brunhil de (Bruni) Schukowski from Eberswalde is 74 years old and already a granny three times over. Last weekend she participated in the Brandenburg People’s Triathlon. The event slogan this year was “Young and Old: Active together” and it was organized by city representatives as well as the Charitas. The competition was also a fundraiser for the newly opened Multigenerational Housing Project “We live together” where Bruni resides.

This example shows that older people are becoming increasingly active. This year, says Bruni, several women and men over 50 were at the start line. In this triathlon “only” the People’s Distance was covered. This is a much shorter distance than that of the professional triathlons.

The first competitors to cross the finish line took about one hour. Competitors were enthusiastically welcomed by children, grandchildren and neighbors. Their participation is good advertisement for the charitable “Living Together Project”. Well, old people, just aren’t what they used to be…

## Supporting information

S1 Data Anonymized collins & clément (2018) data. This anonymized data is available online for public use, as approved by the research ethics board of the University of Ottawa.
(SAV)
